# Dissecting the small RNA code of inflammatory bowel disease

**DOI:** 10.1186/s10020-025-01412-1

**Published:** 2026-02-10

**Authors:** Hawkeye Bufkin Plank, Xudong Zhang, Hukam C. Rawal, Jiancheng Yu, Changcheng Zhou, Qi Chen, Tong Zhou

**Affiliations:** 1 Department of Physiology and Cell Biology, University of Nevada, Reno School of Medicine, Reno, NV 89557 USA; 2 Molecular Medicine Program, Division of Urology, Department of Surgery, University of Utah School of Medicine, Salt Lake City, UT 84132 USA; 3 Department of Human Genetics, University of Utah School of Medicine, Salt Lake City, UT 84132 USA; 4 Division of Biomedical Sciences, School of Medicine, University of California, Riverside, Riverside, CA 92521 USA

**Keywords:** Inflammatory bowel disease, tsRNA, rsRNA, ysRNA, Oxidative stress

## Abstract

**Background:**

Emerging evidence demonstrates that noncanonical small noncoding RNAs (sncRNAs), including tRNA-derived small RNAs (tsRNAs), rRNA-derived small RNAs (rsRNAs), and Y RNA-derived small RNAs (ysRNAs), are highly abundant in various tissues and cell types and play critical roles in numerous biological processes. These noncanonical sncRNAs are also present in bodily fluids with great potential as disease biomarkers.

**Methods:**

We leveraged an existing sncRNA dataset to characterize the profiles of tsRNAs, rsRNAs, and ysRNAs in the peripheral blood of patients with inflammatory bowel disease (IBD)—comprising both ulcerative colitis (UC) and Crohn’s disease (CD)—alongside healthy controls (HCs) and symptomatic controls (SCs) within a Swedish cohort (*n* = 205).

**Results:**

Our analysis revealed an overall similar dysregulation pattern of noncanonical sncRNAs among the UC, CD, and SC samples compared to HCs. Our co-expression analysis between sncRNAs and genes suggests elevated oxidative stress as a potential universal regulator of tsRNA/rsRNA/ysRNA biogenesis in IBD. Further, we developed a molecular signature composed of 21 tsRNA/rsRNA/ysRNA families, which clearly distinguished IBD patients from HCs in the Swedish cohort. However, this signature showed a diminished signal in a German cohort (*n* = 242), in which the IBD patients were treatment-exposed. As a comparison, a 25-miRNA based signature was also developed, which also showed a fairly good classification power between HCs and patients in the Swedish cohort, but was less robust reflected by a stronger baseline divergence between the Swedish and German cohorts.

**Conclusions:**

Our findings suggest the potential of tsRNA/rsRNA/ysRNA profiles as biomarkers for IBD and provide clues for further functional and mechanistic investigations.

**Supplementary Information:**

The online version contains supplementary material available at 10.1186/s10020-025-01412-1.

## Background

Small noncoding RNAs (sncRNAs) have been discovered in both prokaryotes and eukaryotes, and the territory of sncRNAs keeps expanding [1]. In recent years, there has been a wave of technical revolutions in sncRNA sequencing (sncRNA-seq) methods [1] (e.g., PANDORA-seq [2], TGIRT-seq [3], and CPA-seq [4]), along with updated bioinformatic tools [5], leading to the systematic identification of numerous noncanonical sncRNAs that are cleaved from various parental RNAs (e.g., tRNAs, rRNAs, and Y RNAs), such as tRNA-derived small RNAs (tsRNAs), rRNA-derived small RNAs (rsRNAs), and Y RNA-derived small RNAs (ysRNAs) [1, 6, 7]. These sncRNAs carry various RNA modifications and thus could not be systematically discovered using traditional sncRNA-seq methods [1, 2]. Particularly, advances in sncRNA-seq techniques have updated the landscape of sncRNAs, revealing that tsRNAs and rsRNAs are dominant in many tissue and cell types, far exceeding the abundance of miRNAs [2]. This also sets a high standard for future experiments on small RNA profiling using these advanced methods. Noncanonical sncRNAs, such as tsRNAs, rsRNAs, and ysRNAs, have been demonstrated to exert various biological functions in regulating viral infection [8, 9], cancer metastasis [10–12], stem cell differentiation [2, 13–15], neurological diseases [16, 17], cardiovascular function [18–20], immune response [21–23], epigenetic inheritance [24–31], and symbiosis [32, 33]. Importantly, these noncanonical sncRNAs are widely present in bodily fluids and show great potential as biomarkers [6, 17, 34–38] due to their high information capacity, which includes not only the expression levels of individual sequences but also the cleavage patterns/locations from their parental sequences as well as the associated RNA modifications [6].

**Fig. 1:**
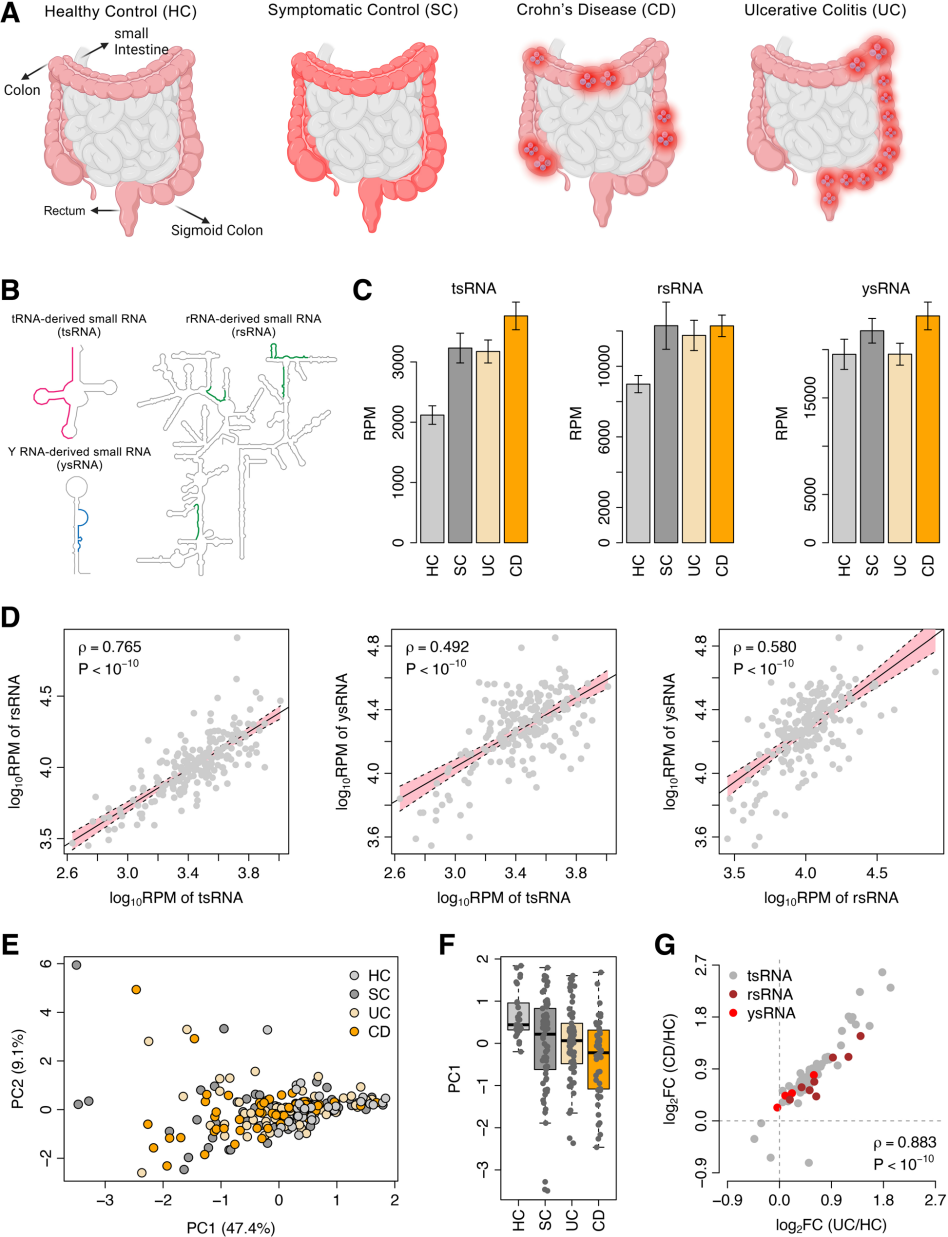
The landscape of noncanonical sncRNAs in IBD. **A** The control/patient categories in the Swedish cohort. [Created in BioRender. Yu, J. (2025) https://BioRender.com/48tdbq0]. **B** Exemplary secondary structures of parental RNAs (i.e., tRNA, rRNA, and Y RNA). The templates of the secondary structures were obtained from RNAcentral (https://rnacentral.org). The regions highlighted in colors indicate the exemplary sncRNAs derived from the parental RNAs. **C** Comparison in total abundance of tsRNAs, rsRNAs, and ysRNAs between the HC, SC, UC, and CD groups. The error bars indicate the standard error of the mean. Compared with HCs, there is a significant increase in tsRNA (*P* < 0.001) and rsRNA (*P* < 0.05) abundance in the SC, UC, and CD groups. There is no significant change in ysRNA abundance between the HC and other groups. **D** The relationship in total abundance between tsRNAs, rsRNAs, and ysRNAs. Both X-axis and Y-axis are log_10_-transformed. The correlation coefficients and *P*-values were calculated using *Spearman*’s rank correlation test. **E** Principal component analysis on noncanonical sncRNA expression. *PC1*: the first principal component; *PC2*: the second principal component. **F** Comparison in *PC1* between the HC, SC, UC, and CD groups. **G** Relationship in expression fold change (*FC*) of noncanonical sncRNA families. Both X-axis and Y-axis are log_2_-transformed. X-axis: the *FC* between the HC and UC groups; Y-axis: the *FC* between the HC and CD groups. The correlation coefficient and *P*-value were calculated using *Spearman*’s rank correlation test

Despite the recent methodological advances in sncRNA sequencing, most existing sncRNA based analyses were performed using traditional sncRNA-seq protocol. While miRNAs constitute the majority of the sequencing reads in these datasets, tsRNAs, rsRNAs, and ysRNAs can be consistently detected in smaller portions. This raises the question of whether these tsRNAs, rsRNAs, and ysRNAs can still be utilized for diagnostic purposes in human diseases and what their performance is compared to miRNAs. To address this knowledge gap, we reanalyzed a previously published sncRNA-seq dataset with relatively large sample size, which was initially used to profile miRNA expression in peripheral blood in inflammatory bowel disease (IBD) by Juzenas et al [39]. IBD is a disorder characterized by chronic inflammation in the digestive tract driven by genetic and environmental factors and associated with certain foods and gut microbiota [40], which can be classified into two subtypes: Crohn’s disease (CD), in which the terminal ileum and colon are most often the source of inflammation, and ulcerative colitis (UC), which is defined by inflammation of the rectum and colon [40] (Fig. 1A). Previously, there were efforts to identify noncoding RNAs as potential biomarkers for IBD but were limited mainly to miRNAs [39, 41–44], and somewhat to long noncoding RNAs and circular RNAs [45, 46]. Though attempts have been made to identify miRNAs differentiating between CD and UC using small cohorts [41, 42], the most recent study conducted by Juzenas et al [39] with comparatively large sample size has not found any significant differences between these two clinical conditions. Although the potential has been discussed [47, 48], to date, no effort has been made to decode the role of other sncRNAs, such as noncanonical sncRNAs (e.g., tsRNAs, rsRNAs, and ysRNAs), in the diagnosis and/or treatment of IBD. Studying these noncanonical sncRNAs in IBD, using the above-mentioned data generated by Juzenas et al [39] with large sample size, may clarify the differences, if any, between CD and UC. In this study, we demonstrated that while miRNAs represented the majority of the sequencing reads in this dataset (with a median percentage of 69.2%), the remaining tsRNA, rsRNA, and ysRNA reads (with a median percentage of 3.4%) could still effectively distinguish between controls and patients. These noncanonical sncRNAs also showed a more robust profile across different cohorts. Further, our co-expression analysis between tsRNAs/rsRNAs/ysRNAs and genes suggests that elevated oxidative stress response could be a common regulator of altered profile of noncanonical sncRNAs in IBD, providing mechanistic insights for future investigation.

## Methods

### Publicly available datasets

The sncRNA-seq data were downloaded from the Gene Expression Omnibus (GEO) database, which were published by [39]. Two datasets were investigated in this study, including the Swedish (GEO accession: GSE169569) and German (GEO accession: GSE169570) cohorts. For both datasets, the libraries were constructed using the Illumina TruSeq Small RNA Sample Preparation Kit and the sequencing was performed using the Illumina HiSeq 2500 System [39]. We also obtained the genome-wide gene expression data of the Swedish cohort from the GEO database (GEO accession: GSE169568), which is based on the Illumina HumanHT-12 V4.0 expression beadchip [39]. The gene expression information summarized by Juzenas et al. is also available from the GitHub website (https://github.com/ikmb/ibd-blood-reproducibility/tree/main/data) [39].

### sncRNA-seq data analysis

The adapters of raw sequencing reads were trimmed by the *Cutadapt* tool (v1.18) [49]. The *SPORTS* framework (v1.1.1) [5] was used to summarize and annotate individual sncRNAs from the trimmed sequencing reads with zero tolerance to mismatch. Only the sncRNA species with length of 15–45 nucleotides (nts) were retained. The “summary” and “output” files generated by *SPORTS* were used to parse the expression information of individual tsRNA/rsRNA/ysRNA families and species, respectively. For tsRNAs, only the species derived from mature parental tRNAs were retained for further analysis. The expression level of the individual sncRNA families and species was measured as reads per million (*RPM*). The differentially expressed sncRNA families were prioritized using a linear regression on *RPM* controlling for age and sex. Only the sncRNA families commonly dysregulated in both the UC and CD groups (adjusted *P* < 0.05) were retained for molecular signature development.

### Prediction and visualization of RNA secondary structure

The *RNAfold* tool [50] was applied to predict the secondary structure of the Y RNA. The secondary structure of the genomic tRNAs was obtained from the GtRNAdb [51, 52], while the secondary structure of the mitochondrial tRNAs was predicted using the *tRNAscan-SE* tool [53, 54]. The visualization of RNA secondary structures was performed using the *forna* tool [55].

### IBD risk score

A scoring scheme was applied to assign each sample a risk score based on the expression of the prioritized upregulated sncRNAs, which is similar to the previous publications [34, 56]. The computational method can be expressed as $\:risk\:score={\sum\:}_{i=1}^{n}({e}_{i}-{\mu\:}_{i})/{\tau\:}_{i}$, in which *e*_*i*_ is the expression (log_2_-transformed [*RPM*+1]) of sncRNA *i* of a given sample and *µ*_*i*_ and *τ*_*i*_ are the mean and standard deviation of sncRNA *i* within the population, respectively [34, 56].

### Pathway analyses

The DAVID Bioinformatics tool [57] was used to identify the KEGG pathways [58] enriched by a given set of genes. For a given pathway, a pathway score was computed using the *FAIME* algorithm [59]. A higher pathway score implies an upregulation in overall expression of the given pathway [59].

### Statistical analyses

All the statistical analyses were performed on the R programming platform. The *Spearman*’s rank correlation test, *t*-test, and linear regression were performed using the “cor.test”, “t.test”, and “lm” functions, respectively. The principal component analysis was performed using the “dudi.pca” function within the “ade4” package. In the cases of multiple-testing, *P*-value adjustment was performed by the “p.adjust” function: the *Bonferroni* correction was applied when identifying differentially expressed sncRNAs in IBD, while the *Benjamini*-*Hochberg* Procedure was used when testing the relationship in expression between sncRNAs and genes. The “roc” function within the “pROC” package was applied to plot the receiver operating characteristic (ROC) curves and to calculate the area under the ROC curve (*AUC*).

## Results

### The landscape of noncanonical sncRNAs in IBD

We obtained the sncRNA-seq data in peripheral blood from the GEO database, which includes two datasets, i.e., the Swedish and German cohorts. There are 30 healthy controls (HCs), 65 symptomatic controls (SCs), 58 UC patients without treatment, and 52 CD patients without treatment in the Swedish cohort, while the German cohort is composed of 65 healthy controls, 77 treatment-exposed UC patients, and 100 treatment-exposed CD patients. First, we applied the computational framework, *SPORTS* [5], to process the trimmed sncRNA-seq data from the Swedish cohort. The *SPORTS* tool can profile not only miRNAs, but also noncanonical sncRNAs, such as tsRNAs, rsRNAs, and ysRNAs (Fig. 1B) from sncRNA-seq data [5]. Compared with the HC samples, we observed a significant increase in tsRNA abundance in the SC, UC, and CD groups, respectively (*t*-test: *P* = 2.4 × 10^− 4^ for SC, *P* = 4.4 × 10^− 5^ for UC, and *P* = 6.7 × 10^− 8^ for CD), as well as a global upregulation of rsRNAs in the SC, UC, CD samples (*t*-test: *P* = 2.3 × 10^− 2^ for SC, *P* = 6.4 × 10^− 3^ for UC, and *P* = 6.4 × 10^− 5^ for CD) (Fig. 1C). However, no significant change was identified for total ysRNA abundance between the HC and other groups (*t*-test: *P* = 0.231 for SC, *P* = 0.994 for UC, and *P* = 0.065 for CD) (Fig. 1C). A positive correlation in total abundance was observed between tsRNAs and rsRNAs (*Spearman*’s rank correlation test: *ρ* = 0.765 and *P* < 10^− 10^), between tsRNAs and ysRNAs (*Spearman*’s rank correlation test: *ρ* = 0.492 and *P* < 10^− 10^), and between rsRNAs and ysRNAs (*Spearman*’s rank correlation test: *ρ* = 0.580 and *P* < 10^− 10^), which suggests a common biogenesis mechanism shared by these noncanonical sncRNAs (Fig. 1D). We further investigated the length distribution of the noncanonical sncRNAs in the HC, SC, UC, and CD groups. Unlike miRNAs, multiple size peaks were observed for tsRNAs, rsRNAs, and ysRNAs. For tsRNAs, two major peaks were identified at the length of 17 nts and 22 nts (Supplementary Fig. S1A), while for ysRNAs, two major peaks were observed at the length of 27 nts and 32 nts (Supplementary Fig. S1B); the length distribution of rsRNAs was even more complicated with several peaks at 15 nts, 17 nts, 23 nts, 26 nts, and 33 nts (Supplementary Fig. S1C). As the tsRNAs, rsRNAs and ysRNAs have specific length peaks and the length distributions are similar across the four groups (HC, SC, UC, and CD), the specific fragmentation pattern of tRNA, rRNAs, and Y RNAs could contain biological information representing specific cellular environment that is responsible for their biogenesis.

We further stratified the tsRNA/rsRNA/ysRNA species according to their parental RNAs. In total, 8 rsRNA categories, 4 ysRNA categories, 48 genomic tsRNA (GtsRNA) categories, and 22 mitochondrial tsRNA categories (MtsRNA) were investigated (Supplementary Table S1). Principal component analysis on the expression of these noncanonical sncRNA categories demonstrated a distinct expression profile between the HC and the other groups (Fig. 1E) and a significant difference in the first principal component (*PC1*) was observed between the HC and SC groups (*t*-test: *P* = 2.2 × 10^− 4^), between the HC and UC groups (*t*-test: *P* = 6.5 × 10^− 5^) and between the HC and CD groups (*t*-test: *P* = 2.4 × 10^− 8^) (Fig. 1F). In addition, we observed a significant but slight difference in *PC1* between the UC and CD groups (*t*-test: *P* = 3.7 × 10^− 2^) (Fig. 1F). To understand how consistent the sncRNA dysregulation pattern is between the UC and CD groups, we calculated the expression fold change (*FC*) for all the tsRNA/rsRNA/ysRNA families between the HC and UC samples and between the HC and CD samples, respectively. A strong positive correlation in log_2_-transformed *FC* was observed (*Spearman*’s rank correlation test: *ρ* = 0.883 and *P* < 10^− 10^) (Fig. 1G), which suggest a highly similar mechanism on tsRNA/rsRNA/ysRNA biogenesis between UC and CD.

### The differentially expressed tsRNAs, rsRNAs, and ysRNAs in IBD

**Fig. 2:**
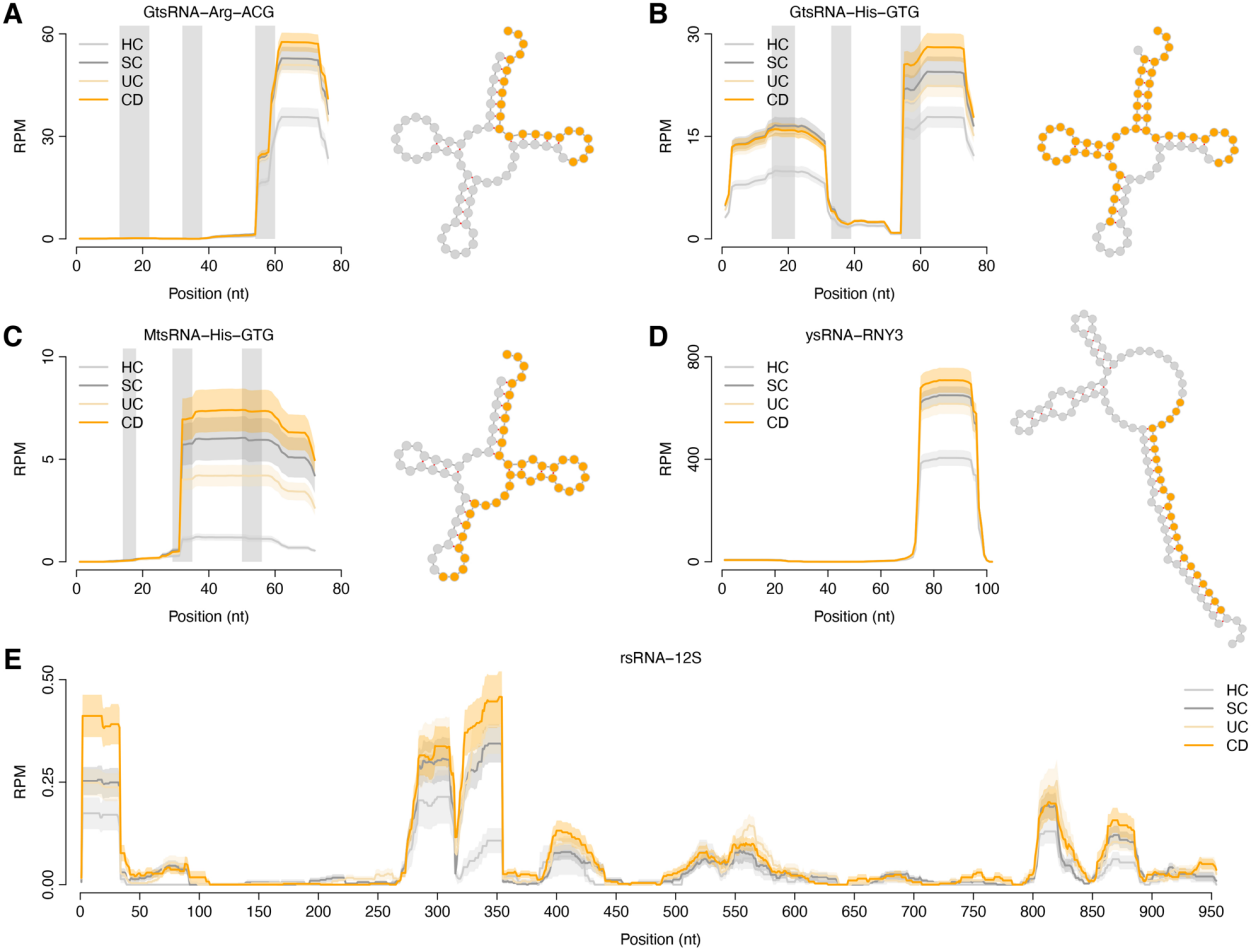
Cleavage pattern of exemplary parental RNAs in the Swedish cohort. The parental RNAs of five exemplary upregulated noncanonical sncRNA families in IBD were plotted, including (**A**) GtsRNA-Arg-ACG, (**B**) GtsRNA-His-GTG, (**C**) MtsRNA-His-GTG, (**D**) ysRNA-RNY3, and (**E**) rsRNA-12S. The X-axis represents the RNA sequences from the 5’ end to the 3’ end. The solid lines indicate the mean in *RPM* while the shaded areas indicate the standard error of the mean. The highlighted nucleotides (in orange) in the RNA secondary structures indicate the primary locations from where the sncRNA species are derived. Three vertical grey bars (panels **A**, **B**, and **C**) from left to right indicate the tRNA positions of D-loop, anticodon-loop and T-loop, respectively

Since Fig. 1G demonstrated a similar dysregulation pattern for all the tsRNA/rsRNA/ysRNA families between UC and CD in the Swedish cohort, here we further statistically investigated the differentially expressed tsRNA/rsRNA/ysRNA families using a linear model controlling for age and sex. In total, 21 noncanonical sncRNA families were identified to be commonly upregulated in both the UC and CD samples compared to the HC group (adjusted *P* < 0.05), including one rsRNA (rsRNA-12S), one ysRNA (ysRNA-RNY3), fifteen GtsRNAs (e.g., GtsRNA-Arg-ACG and GtsRNA-His-GTG), and four MtsRNAs (e.g., MtsRNA-His-GTG) (Fig. 2, Supplementary Fig. S2, and Supplementary Table S2). Interestingly, upregulation of these 21 noncanonical sncRNA families was also identified in the SC group relative to the HCs (*P* < 0.05). We designated these 21 noncanonical sncRNA families as the 21-tsRNA/rsRNA/ysRNA signature.

We also evaluated the classification power of tsRNAs/rsRNAs/ysRNAs in the Swedish cohort, using 1,000 rounds of five-fold cross-validation. In each round of cross-validation, the HC, UC, and CD samples in the Swedish cohort were split into five equal partitions. One partition was used for validation while the remaining partitions were used for training. The training procedure is the same as what we did to identify the 21-tsRNA/rsRNA/ysRNA signature, i.e., prioritizing the commonly dysregulated tsRNA/rsRNA/ysRNA families in both the UC and CD samples (adjusted *P* < 0.05) in the training partitions. A risk score was calculated based on the prioritized tsRNA/rsRNA/ysRNA families in the validation partition (see Methods for details). A greater risk score implies an increased likelihood or severity of IBD. The classification power of the risk score was evaluated by computing the *AUC* between the HC and UC samples and between the HC and CD samples, respectively. This training-validation procedure was repeated five times with each partition used once for validation. A mean *AUC* was subsequently computed for each round of five-fold cross-validation. Supplementary Figure S3A demonstrated that the mean *AUC* ranged from 0.710 to 0.877 with a median of 0.796 for the classification between HC and UC and the mean *AUC* ranged from 0.710 to 0.940 with a median of 0.874 for the classification between HC and CD. In addition, almost all the sncRNA families within the 21-tsRNA/rsRNA/ysRNA signature are most likely to be prioritized during the 1,000 rounds of cross-validation (Supplementary Fig. S3B). All these results suggest the promising diagnostic power of tsRNAs/rsRNAs/ysRNAs in IBD.

### The co-expression between noncanonical sncRNAs and genes

**Fig. 3:**
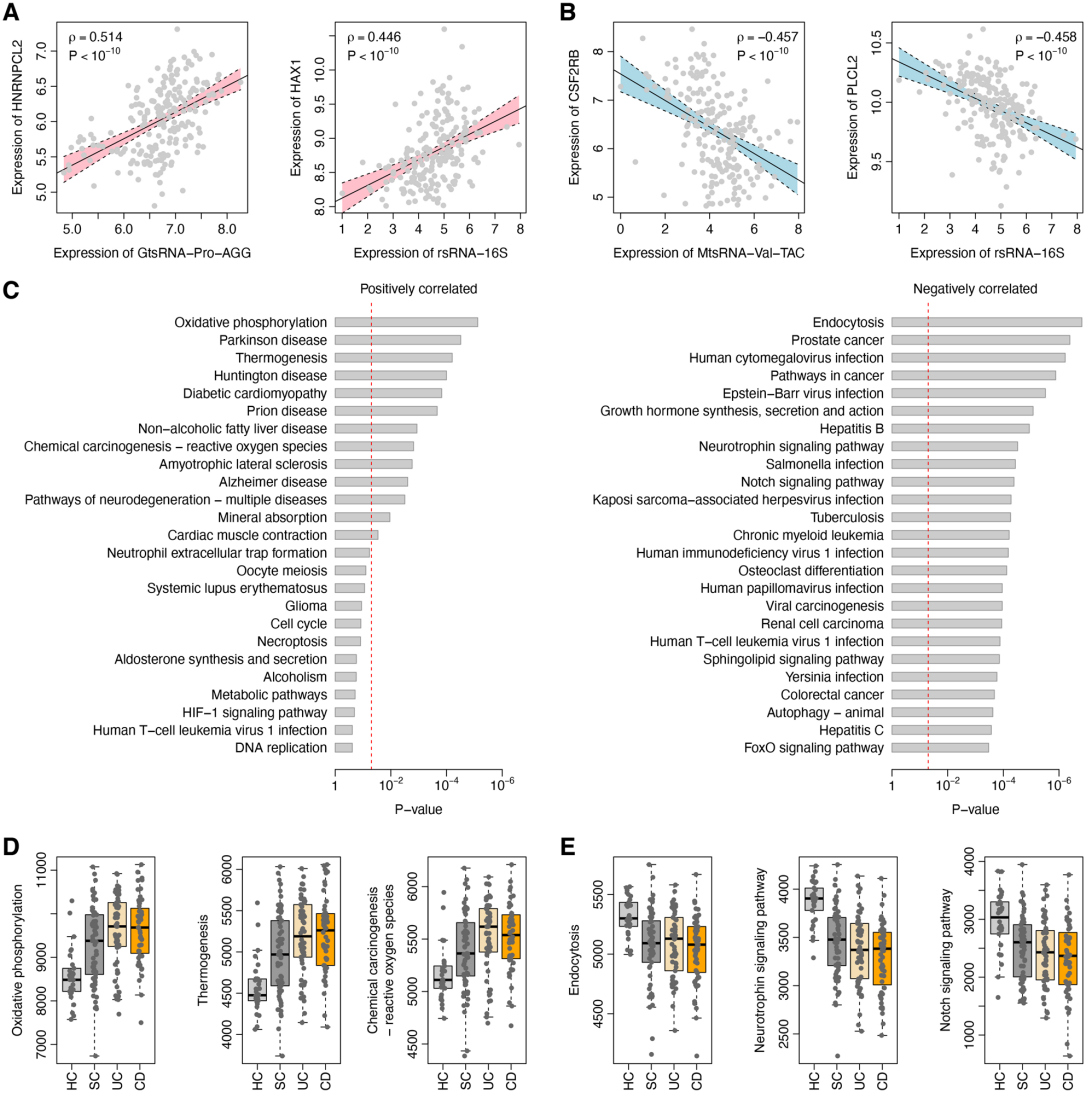
Co-expression between noncanonical sncRNAs and genes. **A** The top positively co-expressed tsRNA-gene and rsRNA-gene pairs. The expression of GtsRNA-Pro-AGG (left panel) and rsRNA-16S (right panel) was positively correlated with that of genes *HNRNPCL2* and *HAX1*, respectively. **B** The top negatively co-expressed tsRNA-gene and rsRNA-gene pairs. The expression of MtsRNA-Val-TAC (left panel) and rsRNA-16S (right panel) was negatively correlated with that of genes *CSF2RB* and *PLCL2*, respectively. The X-axes in (**A**) and (**B**) indicate the values of log_2_-transformed (*RPM* + 1) of the individual sncRNAs while the Y-axes indicate the log_2_-transformed expression intensity of the individual genes. The correlation coefficients and *P*-values were calculated using *Spearman*’s rank correlation test. **C** The top 25 KEGG pathways enriched by the genes co-expressed with noncanonical sncRNAs. The red dash lines indicate the significance level of *α* = 0.05. **D** Comparison in pathway score of the exemplary pathways positively co-expressed with noncanonical sncRNAs. The Y-axes indicate the pathway scores. The pathway scores of these pathways were increased in the SC and IBD samples compared to the HCs (*t*-test: *P* = 2.3 × 10^− 5^ between HC and SC, *P* = 3.8 × 10^− 9^ between HC and UC, and *P* = 1.2 × 10^− 9^ between HC and CD for “Oxidative phosphorylation”; *P* = 1.4 × 10^− 5^ between HC and SC, *P* = 2.4 × 10^− 10^ between HC and UC, and *P* = 3.8 × 10^− 10^ between HC and CD for “Thermogenesis”; *P* = 9.7 × 10^− 4^ between HC and SC, *P* = 3.1 × 10^− 7^ between HC and UC, and *P* = 5.1 × 10^− 7^ between HC and CD for “Chemical carcinogenesis − reactive oxygen species”). **E** Comparison in pathway score of the exemplary pathways negatively co-expressed with noncanonical sncRNAs. The Y-axes indicate the pathway scores. The pathway scores of these pathways were decreased in the SC and IBD groups (*t*-test: *P* = 1.1 × 10^− 6^ between HC and SC, *P* = 1.8 × 10^− 6^ between HC and UC, and *P* = 1.8 × 10^− 7^ between HC and CD for “Endocytosis”; *P* = 5.0 × 10^− 10^ between HC and SC, *P* < 10^− 10^ between HC and UC, and *P* < 10^− 10^ between HC and CD for “Neurotrophin signaling pathway”; *P* = 1.9 × 10^− 4^ between HC and SC, *P* = 8.1 × 10^− 6^ between HC and UC, and *P* = 3.3 × 10^− 6^ between HC and CD for “Notch signaling pathway”)

To systematically understand the relationship between noncanonical sncRNAs and transcriptomic profile, we investigated the co-expression pattern between the tsRNAs/rsRNAs/ysRNAs and genes in the Swedish cohort. Using *Spearman*’s rank correlation test, the pairwise relationship in expression between individual tsRNA/rsRNA/ysRNA families and genes was calculated (Additional file 2). The top sncRNA-gene pairs with the strongest correlation are shown in Fig. 3A and B and Supplementary Fig. S4: GtsRNA-Pro-AGG, rsRNA-16S, and ysRNA-RNY3 were positively co-expressed with *HNRNPCL2*, *HAX1*, and SCX (which are involved in transcription, RNA binding, and ribosome assembly [60–62], respectively; whereas MtsRNA-Val-TAC, rsRNA-16S, and ysRNA-RNY3 were negatively co-expressed with *CSF2RB*, *PLCL2*, and *SNX1* (which are involved in inflammation, autoimmune diseases, and endosome-to-lysosome molecular trafficking [63–65], respectively. We further investigated the pathways enriched by the genes that were co-expressed with tsRNAs/rsRNAs/ysRNAs. Here, only the genes, either positively or negatively co-expressed, with at least 20 sncRNA families were considered. In total, 204 and 755 genes were identified to be positively and negatively co-expressed with tsRNAs/rsRNAs/ysRNAs, respectively. The positively co-expressed genes were significantly associated with the KEGG pathways related to response to oxidative stress, including “Oxidative phosphorylation” and “Chemical carcinogenesis − reactive oxygen species” (Fig. 3C) [66], where the elevation of oxidative stress is a potential factor to increase tRNA/rRNA fragmentation [67–69]. In contrast, the negatively co-expressed genes were significantly associated with more diversified KEGG pathways, such as “Endocytosis”, “Pathways in cancer”, “Neurotrophin signaling pathway”, “Notch signaling pathway”, “Sphingolipid signaling pathway”, and “FoxO signaling pathway” (Fig. 3C). In addition, we found that the pathway scores of the pathways positively co-expressed with noncanonical sncRNAs were significantly higher in the SC and IBD samples compared to the HCs (Fig. 3D). On the contrary, the pathway scores of the pathways negatively co-expressed with tsRNAs/rsRNAs/ysRNAs were significantly lower in the SC and IBD groups (Fig. 3E).

### Comparison between tsRNAs/rsRNAs/ysRNAs and miRNAs in IBD

**Fig. 4:**
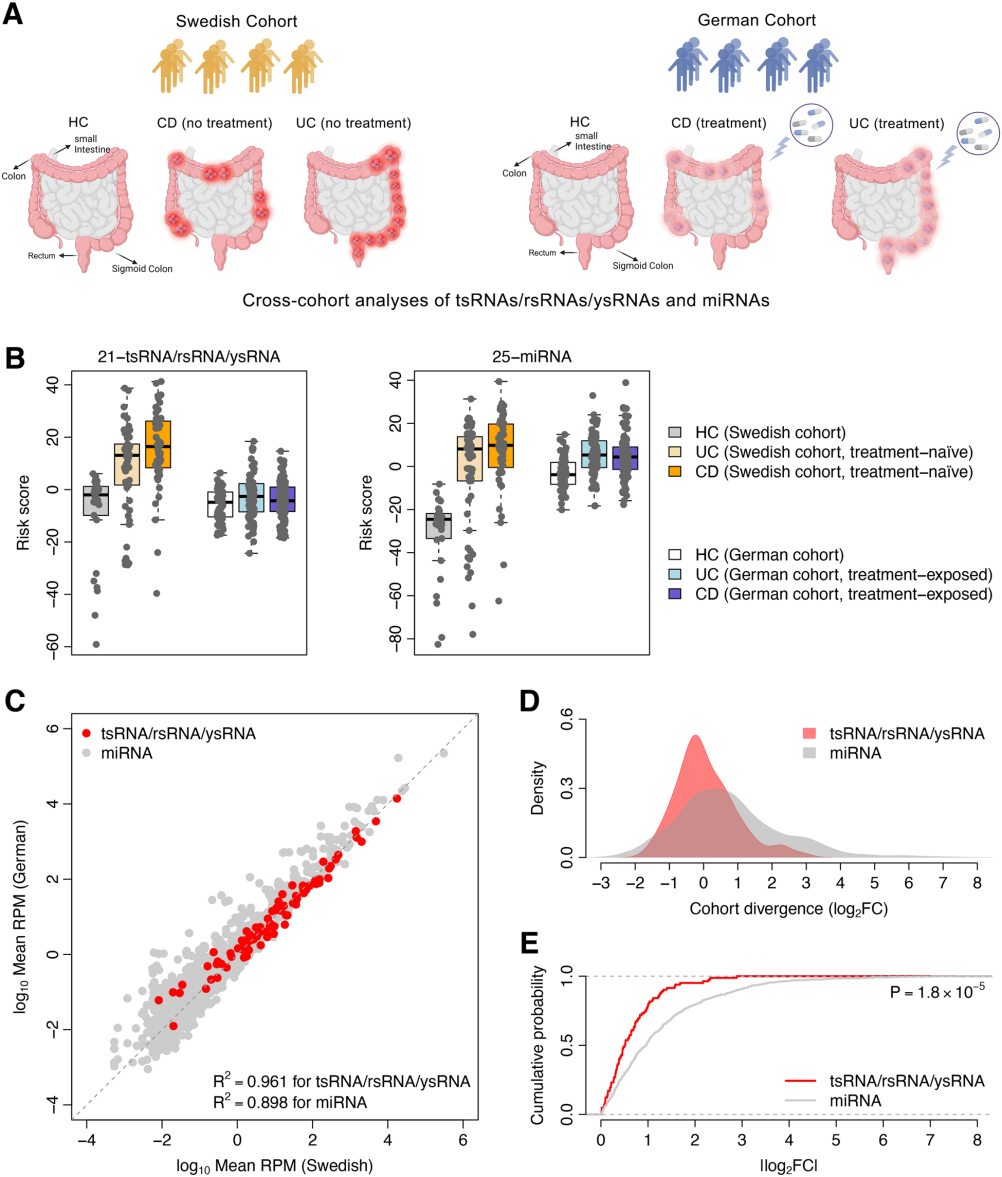
Comparison between tsRNAs/rsRNAs/ysRNAs and miRNAs in IBD. **A** The Swedish and German cohorts. The IBD patients in the Swedish cohort were treatment-naïve while the patients in the German cohort were treatment-exposed. [Created in BioRender. Yu, J. (2025) https://BioRender.com/0frgkku]. **B** Comparison of IBD risk score between the HC, UC, and CD groups. The risk score was calculated based on the 21-tsRNA/rsRNA/ysRNA signature (left panel) and 25-miRNA signature (right panel), respectively. **C** Relationship in tsRNA/rsRNA/ysRNA and miRNA expression between the Swedish and German cohorts. Only the HC samples were considered here. The X-axis and Y-axis indicate the values of log_10_-transformed mean *RPM* in the Swedish and German cohorts, respectively. The *R*^*2*^-values were computed using linear regression between the X-axis and Y-axis. **D** Distributions of cohort divergence. The cohort divergence was computed as the log_2_-transformed fold change in sncRNA expression of the HC samples (log_2_*FC*) between the Swedish and German cohorts. **E **Cumulative distribution of the absolute value of log_2_*FC *(|log_2_*FC*|) of both tsRNAs/rsRNAs/ysRNAs and miRNAs. A significant increase in |log_2_*FC*| was observed for miRNAs relative to tsRNAs/rsRNAs/ysRNAs. The *P*-value was computed using *Kolmogorov-Smirnov* test

First, an IBD risk score was calculated for each sample based on the expression of the 21-tsRNA/rsRNA/ysRNA signature described above (see Methods for details). Both the Swedish and German cohorts were investigated here (Fig. 4A). Compared with the HC samples, the risk score of the IBD samples in the Swedish cohort was significantly higher (*t*-test: *P* = 4.1 × 10^− 5^ for UC and *P* = 2.9 × 10^− 8^ for CD) (Fig. 4B). Also, the risk score of the CD group was slightly but significantly higher than that of the UC group (*t*-test: *P* = 1.6 × 10^− 2^) (Fig. 4B). The ROC curves for group classification revealed that the *AUC* was 0.813 between the HC and UC groups and 0.918 between the HC and CD groups (Supplementary Fig. S5). However, if we looked at the treatment-exposed samples in the German cohort, the risk score of the IBD patients was remarkably recovered to the baseline, though the risk score of the UC samples was slightly higher than that of the HCs (*t*-test: *P* = 1.4 × 10^− 2^) (Fig. 4B). Meanwhile, the German cohort exhibited only two noncanonical sncRNA families (GtsRNA-Arg-ACG and GtsRNA-Gly-TCC, a subset of the 21-tsRNA/rsRNA/ysRNA signature) that were commonly dysregulated in both the UC and CD samples (adjusted *P* < 0.05), which was much less than that of the Swedish cohort (Supplementary Fig. S6). These results indicate that the diagnostic signal of the 21-tsRNA/rsRNA/ysRNA signature was diminished in the patients undergoing treatment.

We further investigated the classification power of the miRNAs in the Swedish cohort. In total, 25 miRNAs were identified to be commonly upregulated or downregulated in both the UC and CD samples compared to the HC group (linear model controlling for age and sex: adjusted *P* < 0.05) (Supplementary Fig. S7), which was designated as the 25-miRNA signature. A risk score was also calculated based on this 25-miRNA signature for each sample. Not surprisingly, the miRNA-based risk score was significantly higher in the IBD patients compared to HCs in the Swedish cohort (*t*-test: *P* = 5.1 × 10^− 8^ for UC and *P* = 1.1 × 10^− 12^ for CD) (Fig. 4B). The *AUC* was 0.834 between the HC and UC groups and 0.942 between the HC and CD groups (Supplementary Fig. S5). There is no significant difference in *AUC* of the ROC curves between the 21-tsRNA/rsRNA/ysRNA and 25-miRNA signatures for the classification of HC vs. UC (*Delong*’s test: *P* = 0.355), while a marginal difference was observed for the classification of HC vs. CD (*Delong*’s test: *P* = 0.037).

Interestingly, when we compared the 25-miRNA based risk score between the Swedish and German cohorts, we noticed that the German cohort exhibited a global shift in baseline score from that of the Swedish cohort (Fig. 4B): the 25-miRNA based risk score of the HC samples in the German cohort was significantly higher than that of the HCs in the Swedish cohort (*t*-test: *P* = 3.5 × 10^− 9^), which suggests a stronger cohort divergence in baseline miRNA expression pattern compared to noncanonical sncRNAs. Moreover, for the HC samples, the relationship in tsRNA/rsRNA/ysRNA expression between the two cohorts was more linear compared with miRNAs (Fig. 4C) and the cohort divergence of miRNAs was much wider than that of the noncanonical sncRNAs (Fig. 4D and E). Thus, the cross-cohort stability observed in the HC samples suggests that the tsRNAs/rsRNAs/ysRNAs may serve as a technically more robust biomarker compared to miRNAs, as the latter exhibited significant baseline shifts between cohorts.

## Discussion

While the genome-wide mRNA and miRNA expression in peripheral blood of IBD has been comprehensively investigated in the previous study by [39], here we focused on a group of noncanonical sncRNAs, including tsRNAs, rsRNAs, and ysRNAs. Systemic upregulation of these noncanonical sncRNAs was observed in the IBD patients, suggesting enhanced biogenesis and potential diagnostic power of tsRNAs/rsRNAs/ysRNAs in IBD.

A significant finding of our study is that although the proportion of tsRNAs, rsRNAs, and ysRNAs (median percentage: 3.4%) in traditional sncRNA-seq datasets is far lower than that of miRNAs (median percentage: 69.2%), they are sufficient to distinguish IBD patients from HCs. Particularly, tsRNAs/rsRNAs/ysRNAs exhibit better robustness in the baseline expression level in the cross-cohort analysis (i.e., the Swedish and German cohorts), whereas the miRNA-based signature shows a significant shift in the baseline level between the two cohorts, highlighting the advantage of using tsRNA/rsRNA/ysRNA-based signatures when multi-center cohorts are involved.

Why are tsRNA/rsRNA/ysRNA profiles more stable across cohorts compared to miRNAs? The answer might lie in their distinct biogenesis pathways. While miRNA biogenesis involves relatively simple and defined enzymes (the nuclear Drosha and the cytoplasmic Dicer), the biogenesis of tsRNAs, rsRNAs, and ysRNAs results from the differential cleavage of their parental RNAs (i.e., tRNA, rRNA, and Y RNA) by a much broader range of RNases (e.g., RNase A, L, T2, and Z) [6, 70]. The RNase cleavage activities are further regulated by site-specific RNA modifications, which are tightly controlled by at least dozens of enzymes [6]. Thus, the ultimate profile of tsRNAs/rsRNAs/ysRNAs represents a complex combination of multiple enzymes, many of which (RNases and RNA modification enzymes) are evolutionarily more ancient than Drosha and Dicer [6, 70]. These ancient enzymes function as the foundation of many biological processes and could be less affected by genetic background in cross-cohort analyses.

Intriguingly, oxidative stress is a key characteristic of IBD [71], and increased oxidative stress has been found to be a potential factor impacting tsRNA/rsRNA/ysRNA biogenesis in both unicellular organisms and mammalian cells, facilitating the cleavage of tRNA/rRNA/Y RNA into fragments [67–69], through various enzymes [6,7]. This resonates with the observed increase in oxidative stress responses in CD, UC, and SC groups and their similarly increased tsRNA/rsRNA/ysRNA profiles in the Swedish cohort. (The SC group may include patients under bacterial infection as described by [39] and thus also shows inflammation and increased oxidative stress.) In the German cohort, the tsRNA/rsRNA/ysRNA profiles in the treated CD and UC groups reverted to normal levels, which could be due to controlled inflammation and oxidative stress, although we lack direct transcriptomic data from the German cohort to support this.

On the other hand, the similar dysregulation of tsRNAs, rsRNAs, and ysRNAs observed within the CD, UC, and SC groups might also be due to methodological limitations in traditional sncRNA-seq, as many highly modified tsRNAs/rsRNAs/ysRNAs could not be included in the sequencing library. It is possible that when more advanced sequencing methods (e.g., PANDORA-seq [2] are utilized, more layers of information from modified tsRNAs/rsRNAs/ysRNAs will be revealed, potentially distinguishing between subgroups of IBD (i.e., CD vs. UC) and the SC group. This direction warrants future investigations. Another limitation of this study lies in the lack of validation of the tsRNA/rsRNA/ysRNA-based signature in an independent cohort. The patients from the German cohort were treatment-exposed, which made the dataset not suitable for validation purpose. Future research in other IBD cohorts may further our understanding regarding the classification performance of the 21-tsRNA/rsRNA/ysRNA signature.

Finally, it remains an interesting question whether the observed change in tsRNA/rsRNA/ysRNA profile in IBD represents merely an outcome of altered cellular changes or this altered sncRNA signature can further trigger the sustained inflammation and progression of IBD. IBD constitutes a group of autoimmune diseases where Toll-like receptor 7/8 (TLR7/8)-mediated innate immune dysfunction is deeply involved [72, 73]. TLR7 and TLR8 are X-linked IBD susceptibility genes [74, 75]. Recent studies have shown that tsRNAs, rsRNAs, and ysRNAs are directly involved in triggering the activation of TLR7/8 [22, 23, 76–78]. The abnormally elevated levels of tsRNAs/rsRNAs/ysRNAs found in the blood of IBD patients suggest that tsRNAs/rsRNAs/ysRNAs might be transported throughout the system via extracellular vesicles (EVs), which are known to carry various tsRNAs/rsRNAs/ysRNAs [6, 79, 80], and may ectopically trigger TLR7/8 to either initiate or sustain the inflammatory state [81]. This idea aligns well with the recent proposal that many tsRNAs/rsRNAs/ysRNAs can exert their functions beyond RNA interference and are independent of Argonaute proteins, instead utilizing their secondary and tertiary structures to interact with various proteins in an aptamer-like fashion [70, 82]. Deeper understanding of these sncRNA-TLR interactions may lead to better mechanistic insights into disease etiology and ultimately novel therapeutic means to treat IBD. Beyond testing blood sncRNAs, future exploration may also detect sncRNAs from other biofluids, or even from feces [83] to examine changes more directly reflecting the gastrointestinal compartment.

## Conclusion

We reveal an overall upregulation of noncanonical sncRNAs among the UC, CD, and SC groups compared to HCs, which was previously unexplored. Intriguingly, although these public datasets were generated by traditional sncRNA-seq protocol with miRNAs as the majority, the remaining tsRNAs/rsRNAs/ysRNAs could still effectively distinguish between healthy controls and IBD patients and outperform miRNAs regarding cross-cohort robustness. Further, our co-expression analysis between tsRNAs/rsRNAs/ysRNAs and genes suggests that elevated oxidative stress response could be a common regulator of the altered profile of noncanonical sncRNAs.

## Supplementary Information

Additional file 1. Supplementary figures and tables Supplementary Figure S1. Length distribution of (A) tsRNAs, (B) ysRNAs, and (C) rsRNAs. The solid lines indicate the mean in *RPM* while the shaded areas indicate the 95% confidence interval. Supplementary Figure S2. Differentially expressed noncanonical sncRNA families in IBD in the Swedish cohort. Y-axis indicates the expression level in *RPM*. Using a linear model controlling for age and sex, 21 tsRNA/rsRNA/ysRNA families were found to be commonly upregulated (adjusted *P* < 0.05) in both the UC and CD samples compared to the HC group. Supplementary Figure S3. Five-fold cross-validation on the classification power of tsRNAs, rsRNAs, and ysRNAs in the Swedish cohort. (A) Histogram of mean *AUC* of 1,000 rounds of five-fold cross-validation. (B) Frequency of tsRNA/rsRNA/ysRNA families prioritized during the 1,000 rounds of cross-validation. In total, only 55 tsRNA/rsRNA/ysRNA families were prioritized at least once. The frequency of the sncRNA families within the 21-tsRNA/rsRNA/ysRNA signature ranges from 347 to 4,842 with a median of 2,515. Supplementary Figure S4. The top positively and negatively co-expressed ysRNA-gene pairs. The expression of ysRNA-RNY3 was positively correlated with that of gene *SCX* (left panel), while the expression of ysRNA-RNY3 was negatively correlated with that of gene*SNX1* (right panel). The X-axes indicate the values of log_2_-transformed (*RPM*+1) of ysRNA-RNY3 while the Y-axes indicate the log_2_-transformed expression intensity of the individual genes. The correlation coefficients and *P*-values were calculated using *Spearman*’s rank correlation test. Supplementary Figure S5. ROC curves for the classification between the HC and UC samples and between the HC and CD samples in the Swedish cohort. Supplementary Figure S6. Correlation in *t*-statistic. For each cohort, differential expression analysis was performed between the HC and UC samples and between the HC and CD samples, respectively, using a linear model controlling for age and sex. Each dot represents one tsRNA/rsRNA/ysRNA family. Positive correlation in *t*-statistic computed by the linear model was observed between the comparison of HC *vs.* UC and the comparison of HC *vs.* CD. The red dots are the commonly dysregulated sncRNA families in both comparisons,*i.e.*, 21 and 2 tsRNA/rsRNA/ysRNA families in the Swedish and German cohorts, respectively. The correlation coefficients and*P*-values were calculated using *Spearman*’s rank correlation test. Supplementary Figure S7. Differentially expressed miRNAs in IBD in the Swedish cohort. Y-axis indicates the miRNA expression in *RPM*. Using a linear model controlling for age and sex, 25 miRNAs were found to be commonly upregulated or downregulated (adjusted *P* < 0.05) in both the UC and CD samples compared to the HC group. Supplementary Table S1. Noncanonical sncRNA families Supplementary Table S2. Noncanonical sncRNA families commonly upregulated in the UC and CD samples in the Swedish cohort.

Additional file 2. *Spearman*’s rank correlation in expression between individual noncanonical sncRNA families and genes.

Additional file 3. Key data and code for reproducibility purposes.

## Data Availability

The transcriptomic and sncRNA-seq data were generated by Juzenas et al., which are publicly available from the GEO database (GSE169568, GSE169569, and GSE169570) and the GitHub website (https://github.com/ikmb/ibd-blood-reproducibility/tree/main/data). The key data and code for reproducibility purposes are included in Additional file 3.

## Abbreviations

sncRNA: Small noncoding RNAs
sncRNA-seq: sncRNA sequencing
tsRNA: tRNA-derived small RNAs
rsRNA: rRNA-derived small RNAs
ysRNA: Y RNA-derived small RNAs
GtsRNA: Genomic tsRNA
MtsRNA: Mitochondrial tsRNA
RPM: Reads per million
IBD: Inflammatory bowel disease
UC: Ulcerative colitis
CD: Crohn’s disease
HC: Healthy controls
SC: Symptomatic controls
GEO: Gene Expression Omnibus
FC: Fold change
nt: Nucleotide
ROC: Receiver operating characteristic
AUC: The area under the ROC curve
