# Supplementary material for: Dissecting the small RNA code of inflammatory bowel disease
Source: Mol Med. 2026 Feb 10;32:21. doi: 10.1186/s10020-025-01412-1 (PMC12892614; doi:10.1186/s10020-025-01412-1)
Supplement: Supplementary file 1 — Additional file 1. Supplementary figures and tables Supplementary Figure S1. Length distribution of (A) tsRNAs, (B) ysRNAs, and (C) rsRNAs. The solid lines indicate the mean in RPM while the shaded areas indicate the 95% confidence interval. Supplementary Figure S2. Differentially expressed noncanonical sncRNA families in IBD in the Swedish cohort. Y-axis indicates the expression level in RPM. Using a linear model controlling for age and sex, 21 tsRNA/rsRNA/ysRNA families were found to be commonly upregulated (adjusted P < 0.05) in both the UC and CD samples compared to the HC group. Supplementary Figure S3. Five-fold cross-validation on the classification power of tsRNAs, rsRNAs, and ysRNAs in the Swedish cohort. (A) Histogram of mean AUC of 1,000 rounds of five-fold cross-validation. (B) Frequency of tsRNA/rsRNA/ysRNA families prioritized during the 1,000 rounds of cross-validation. In total, only 55 tsRNA/rsRNA/ysRNA families were prioritized at least once. The frequency of the sncRNA families within the 21-tsRNA/rsRNA/ysRNA signature ranges from 347 to 4,842 with a median of 2,515. Supplementary Figure S4. The top positively and negatively co-expressed ysRNA-gene pairs. The expression of ysRNA-RNY3 was positively correlated with that of gene SCX (left panel), while the expression of ysRNA-RNY3 was negatively correlated with that of geneSNX1 (right panel). The X-axes indicate the values of log2-transformed (RPM+1) of ysRNA-RNY3 while the Y-axes indicate the log2-transformed expression intensity of the individual genes. The correlation coefficients and P-values were calculated using Spearman’s rank correlation test. Supplementary Figure S5. ROC curves for the classification between the HC and UC samples and between the HC and CD samples in the Swedish cohort. Supplementary Figure S6. Correlation in t-statistic. For each cohort, differential expression analysis was performed between the HC and UC samples and between the HC and CD samples, respectively, using a line [file 10020_2025_1412_MOESM1_ESM.pdf]

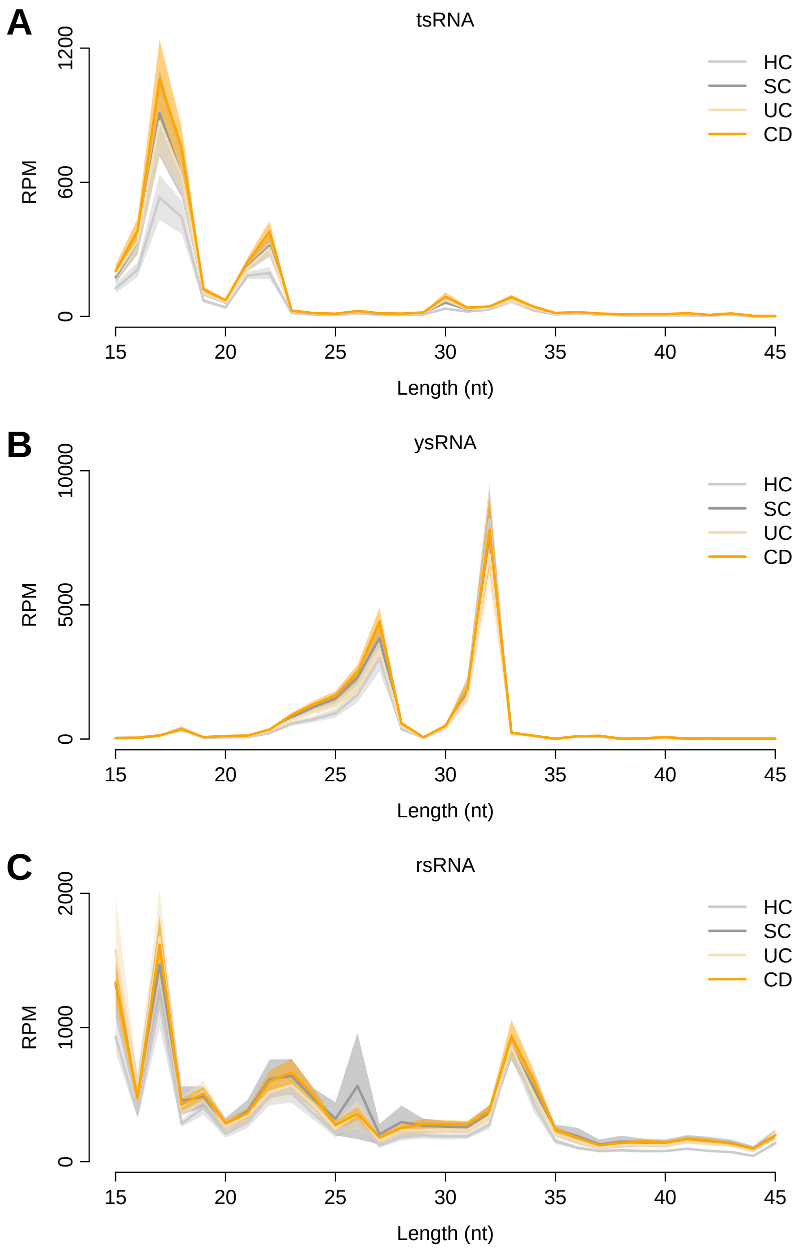

Supplementary Figure S1. Length distribution of (A) tsRNAs, (B) ysRNAs, and (C) rsRNAs. The solid lines indicate the mean in RPM while the shaded areas indicate the 95% confidence interval.

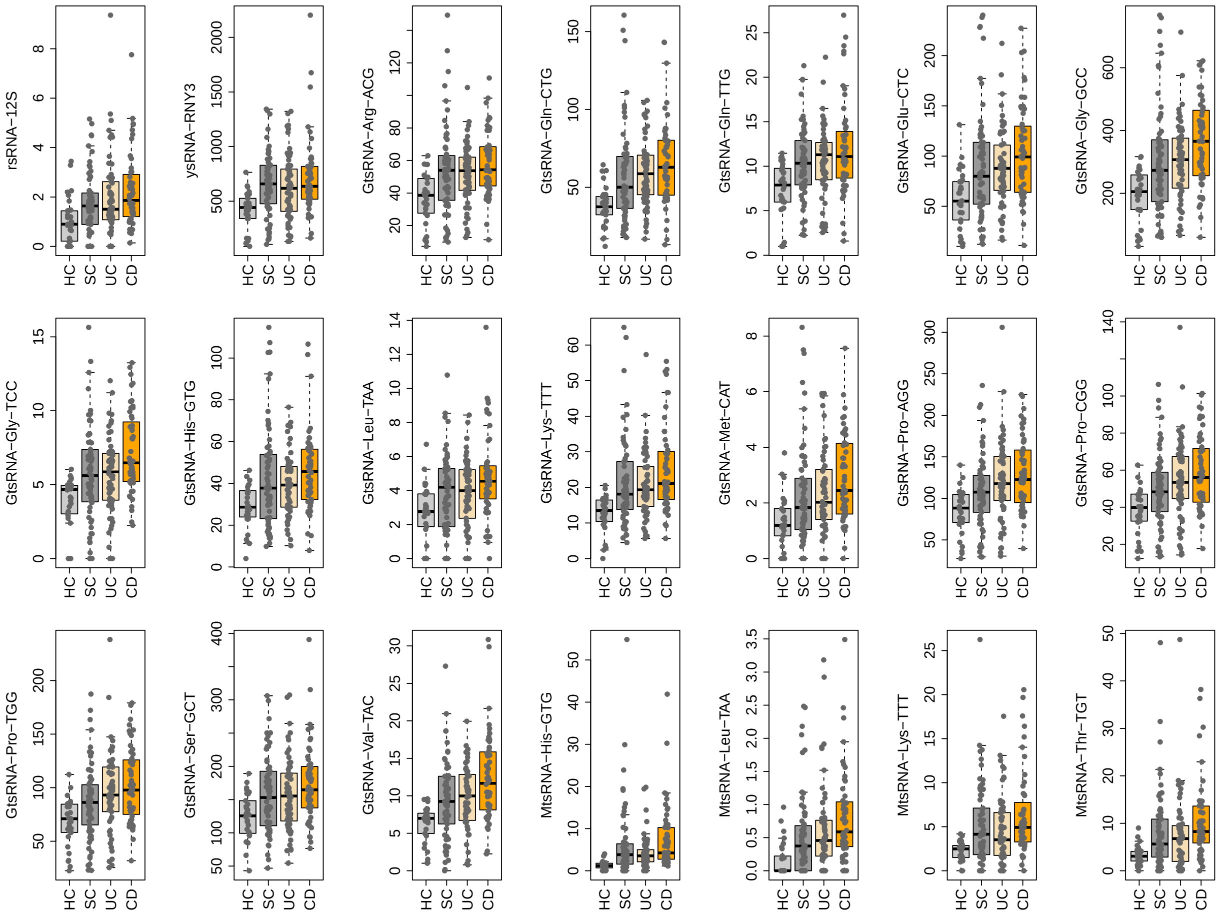

Supplementary Figure S2. Differentially expressed noncanonical sncRNA families in IBD in the Swedish cohort. Y-axis indicates the expression level in RPM. Using a linear model controlling for age and sex, 21 tsRNA/rsRNA/ysRNA families were found to be commonly upregulated (adjusted $P < 0.05$) in both the UC and CD samples compared to the HC group.

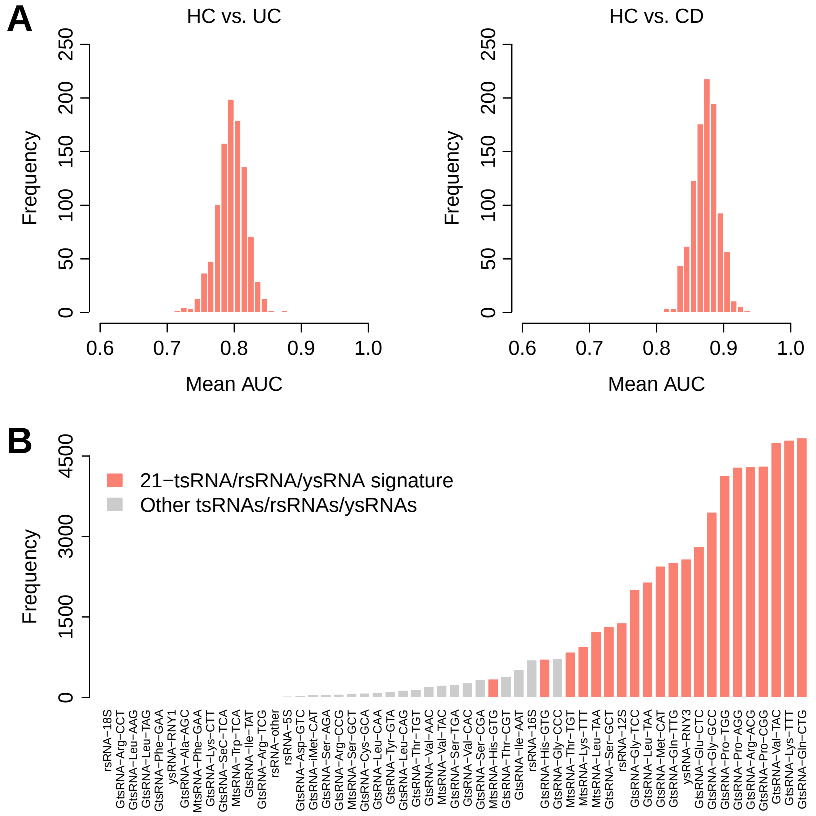

Supplementary Figure S3. Five-fold cross-validation on the classification power of tsRNAs, rsRNAs, and ysRNAs in the Swedish cohort. (A) Histogram of mean AUC of 1,000 rounds of five-fold cross-validation. (B) Frequency of tsRNA/rsRNA/ysRNA families prioritized during the 1,000 rounds of cross-validation. In total, only 55 tsRNA/rsRNA/ysRNA families were prioritized at least once. The frequency of the sncRNA families within the 21-tsRNA/rsRNA/ysRNA signature ranges from 347 to 4,842 with a median of 2,515.

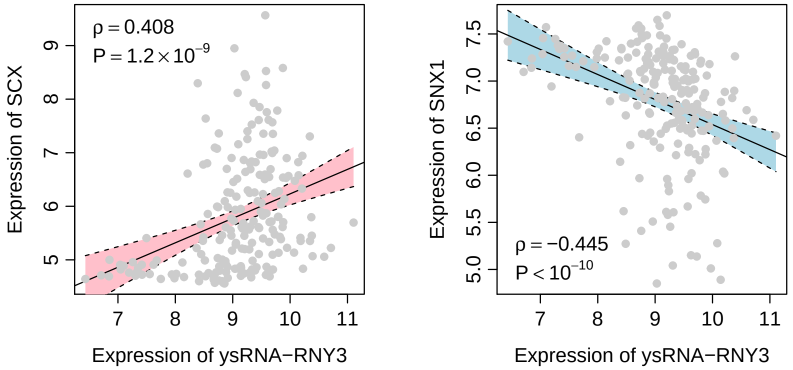

Supplementary Figure S4. The top positively and negatively co-expressed ysRNA-gene pairs. The expression of ysRNA-RNY3 was positively correlated with that of gene *SCX* (left panel), while the expression of ysRNA-RNY3 was negatively correlated with that of gene *SNX1* (right panel). The X-axes indicate the values of log_2-transformed (RPM+1) of ysRNA-RNY3 while the Y-axes indicate the log_2-transformed expression intensity of the individual genes. The correlation coefficients and P-values were calculated using *Spearman*'s rank correlation test.

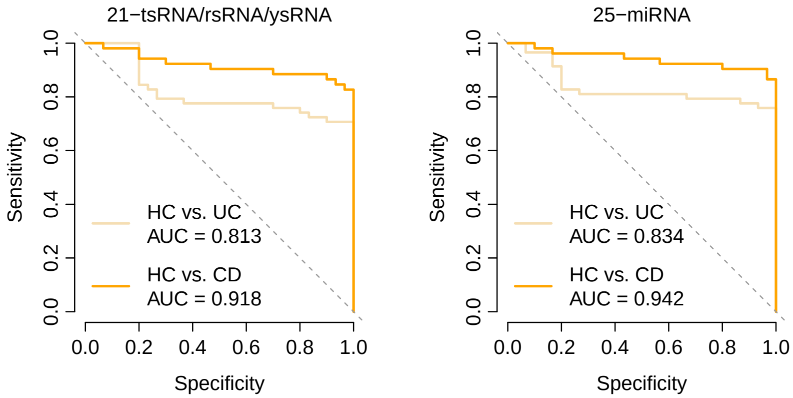

Supplementary Figure S5. ROC curves for the classification between the HC and UC samples and between the HC and CD samples in the Swedish cohort.

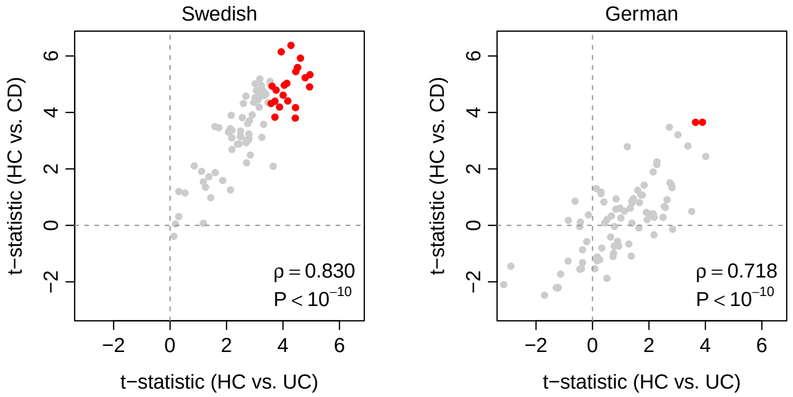

Supplementary Figure S6. Correlation in *t*-statistic. For each cohort, differential expression analysis was performed between the HC and UC samples and between the HC and CD samples, respectively, using a linear model controlling for age and sex. Each dot represents one tsRNA/rsRNA/ysRNA family. Positive correlation in *t*-statistic computed by the linear model was observed between the comparison of HC vs. UC and the comparison of HC vs. CD. The red dots are the commonly dysregulated sncRNA families in both comparisons, *i.e.*, 21 and 2 tsRNA/rsRNA/ysRNA families in the Swedish and German cohorts, respectively. The correlation coefficients and *P*-values were calculated using *Spearman*'s rank correlation test.

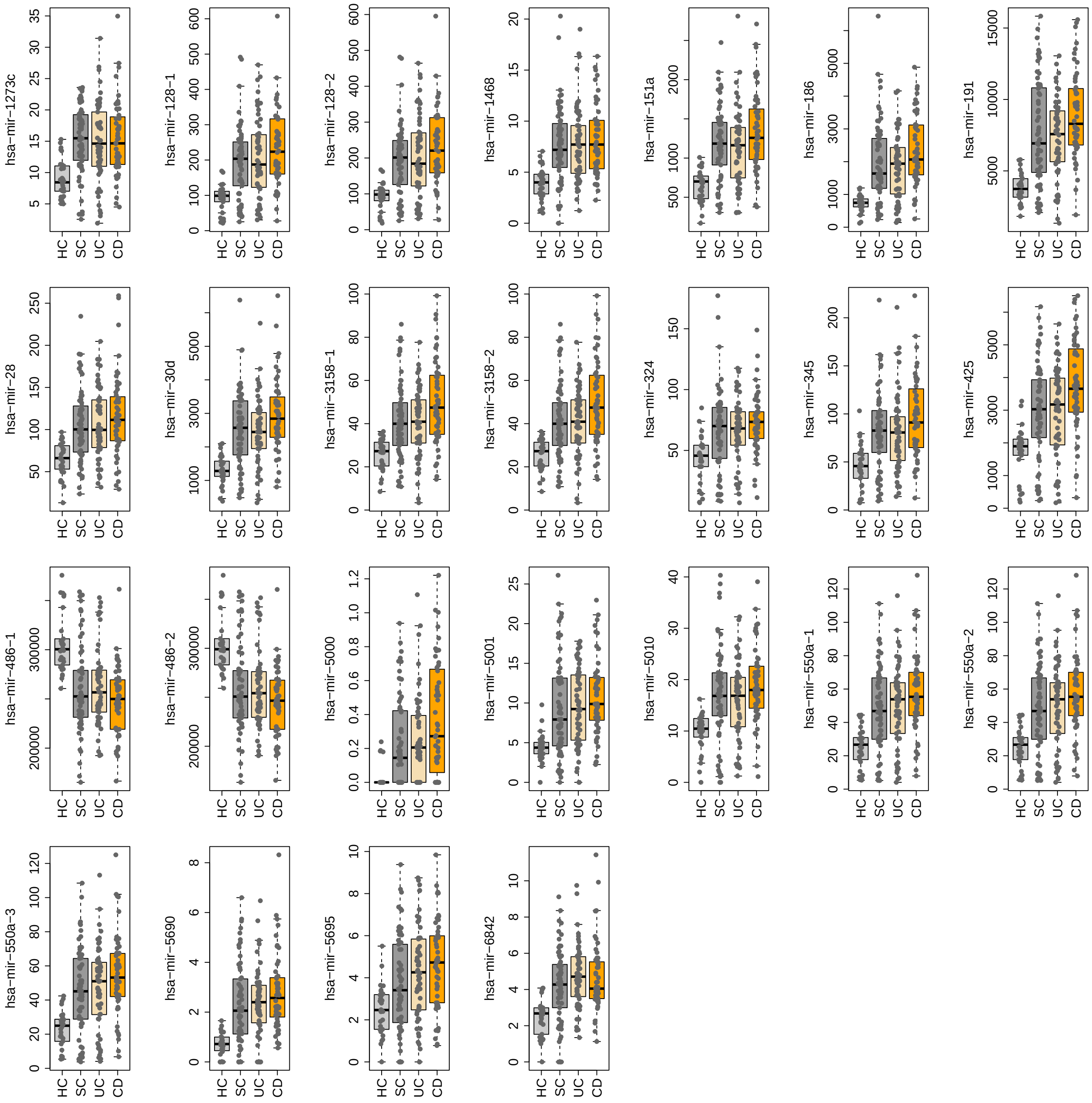

Supplementary Figure S7. Differentially expressed miRNAs in IBD in the Swedish cohort. Y-axis indicates the miRNA expression in RPM. Using a linear model controlling for age and sex, 25 miRNAs were found to be commonly upregulated or down-regulated (adjusted $P < 0.05$) in both the UC and CD samples compared to the HC group.

Supplementary Table S1. Noncanonical sncRNA families

Parental RNA	sncRNA family
12S-rRNA	rsRNA-12S
16S-rRNA	rsRNA-16S
18S-rRNA	rsRNA-18S
28S-rRNA	rsRNA-28S
45S-rRNA	rsRNA-45S
5.8S-rRNA	rsRNA-5.8S
5S-rRNA	rsRNA-5S
other-rRNA	rsRNA-other
RNY1-YRNA	ysRNA-RNY1
RNY3-YRNA	ysRNA-RNY3
RNY4-YRNA	ysRNA-RNY4
RNY5-YRNA	ysRNA-RNY5
Genomic tRNA-Ala-AGC	GtsRNA-Ala-AGC
Genomic tRNA-Ala-CGC	GtsRNA-Ala-CGC
Genomic tRNA-Ala-TGC	GtsRNA-Ala-TGC
Genomic tRNA-Arg-ACG	GtsRNA-Arg-ACG
Genomic tRNA-Arg-CCG	GtsRNA-Arg-CCG
Genomic tRNA-Arg-CCT	GtsRNA-Arg-CCT
Genomic tRNA-Arg-TCG	GtsRNA-Arg-TCG
Genomic tRNA-Arg-TCT	GtsRNA-Arg-TCT
Genomic tRNA-Asn-GTT	GtsRNA-Asn-GTT
Genomic tRNA-Asp-GTC	GtsRNA-Asp-GTC
Genomic tRNA-Cys-GCA	GtsRNA-Cys-GCA
Genomic tRNA-Gln-CTG	GtsRNA-Gln-CTG
Genomic tRNA-Gln-TTG	GtsRNA-Gln-TTG
Genomic tRNA-Glu-CTC	GtsRNA-Glu-CTC
Genomic tRNA-Glu-TTC	GtsRNA-Glu-TTC
Genomic tRNA-Gly-CCC	GtsRNA-Gly-CCC
Genomic tRNA-Gly-GCC	GtsRNA-Gly-GCC
Genomic tRNA-Gly-TCC	GtsRNA-Gly-TCC
Genomic tRNA-His-GTG	GtsRNA-His-GTG
Genomic tRNA-Ile-AAT	GtsRNA-Ile-AAT
Genomic tRNA-Ile-GAT	GtsRNA-Ile-GAT
Genomic tRNA-Ile-TAT	GtsRNA-Ile-TAT
Genomic tRNA-iMet-CAT	GtsRNA-iMet-CAT
Genomic tRNA-Leu-AAG	GtsRNA-Leu-AAG
Genomic tRNA-Leu-CAA	GtsRNA-Leu-CAA
Genomic tRNA-Leu-CAG	GtsRNA-Leu-CAG
Genomic tRNA-Leu-TAA	GtsRNA-Leu-TAA
Genomic tRNA-Leu-TAG	GtsRNA-Leu-TAG
Genomic tRNA-Lys-CTT	GtsRNA-Lys-CTT
Genomic tRNA-Lys-TTT	GtsRNA-Lys-TTT

Parental RNA	sncRNA family
Genomic tRNA-Met-CAT	GtsRNA-Met-CAT
Genomic tRNA-Phe-GAA	GtsRNA-Phe-GAA
Genomic tRNA-Pro-AGG	GtsRNA-Pro-AGG
Genomic tRNA-Pro-CGG	GtsRNA-Pro-CGG
Genomic tRNA-Pro-TGG	GtsRNA-Pro-TGG
Genomic tRNA-SeC-TCA	GtsRNA-SeC-TCA
Genomic tRNA-Ser-AGA	GtsRNA-Ser-AGA
Genomic tRNA-Ser-CGA	GtsRNA-Ser-CGA
Genomic tRNA-Ser-GCT	GtsRNA-Ser-GCT
Genomic tRNA-Ser-TGA	GtsRNA-Ser-TGA
Genomic tRNA-Thr-AGT	GtsRNA-Thr-AGT
Genomic tRNA-Thr-CGT	GtsRNA-Thr-CGT
Genomic tRNA-Thr-TGT	GtsRNA-Thr-TGT
Genomic tRNA-Trp-CCA	GtsRNA-Trp-CCA
Genomic tRNA-Tyr-GTA	GtsRNA-Tyr-GTA
Genomic tRNA-Val-AAC	GtsRNA-Val-AAC
Genomic tRNA-Val-CAC	GtsRNA-Val-CAC
Genomic tRNA-Val-TAC	GtsRNA-Val-TAC
Mitochondrial tRNA-Ala-TGC	MtsRNA-Ala-TGC
Mitochondrial tRNA-Arg-TCG	MtsRNA-Arg-TCG
Mitochondrial tRNA-Asn-GTT	MtsRNA-Asn-GTT
Mitochondrial tRNA-Asp-GTC	MtsRNA-Asp-GTC
Mitochondrial tRNA-Cys-GCA	MtsRNA-Cys-GCA
Mitochondrial tRNA-Gln-TTG	MtsRNA-Gln-TTG
Mitochondrial tRNA-Glu-TTC	MtsRNA-Glu-TTC
Mitochondrial tRNA-Gly-TCC	MtsRNA-Gly-TCC
Mitochondrial tRNA-His-GTG	MtsRNA-His-GTG
Mitochondrial tRNA-Ile-GAT	MtsRNA-Ile-GAT
Mitochondrial tRNA-Leu-TAA	MtsRNA-Leu-TAA
Mitochondrial tRNA-Leu-TAG	MtsRNA-Leu-TAG
Mitochondrial tRNA-Lys-TTT	MtsRNA-Lys-TTT
Mitochondrial tRNA-Met-CAT	MtsRNA-Met-CAT
Mitochondrial tRNA-Phe-GAA	MtsRNA-Phe-GAA
Mitochondrial tRNA-Pro-TGG	MtsRNA-Pro-TGG
Mitochondrial tRNA-Ser-GCT	MtsRNA-Ser-GCT
Mitochondrial tRNA-Ser-TGA	MtsRNA-Ser-TGA
Mitochondrial tRNA-Thr-TGT	MtsRNA-Thr-TGT
Mitochondrial tRNA-Trp-TCA	MtsRNA-Trp-TCA
Mitochondrial tRNA-Tyr-GTA	MtsRNA-Tyr-GTA
Mitochondrial tRNA-Val-TAC	MtsRNA-Val-TAC

Supplementary Table S2. Noncanonical sncRNA families commonly upregulated in the UC and CD samples in the Swedish cohort

	SC		UC		CD	
sncRNA family	*t*-statistic	*P*	*t*-statistic	Adjusted *P*	*t*-statistic	Adjusted *P*
rsRNA-12S	2.550	1.2×10^{-2}	4.436	2.3×10^{-3}	3.800	2.3×10^{-2}
ysRNA-RNY3	4.511	1.9×10^{-5}	4.443	2.2×10^{-3}	4.171	6.4×10^{-3}
GtsRNA-Arg-ACG	3.228	1.7×10^{-3}	4.619	1.1×10^{-3}	5.923	6.6×10^{-6}
GtsRNA-Gln-CTG	3.090	2.7×10^{-3}	4.955	3.0×10^{-4}	5.337	7.4×10^{-5}
GtsRNA-Gln-TTG	3.919	1.7×10^{-4}	4.170	6.1×10^{-3}	4.407	2.7×10^{-3}
GtsRNA-Glu-CTC	2.861	5.2×10^{-3}	4.137	6.8×10^{-3}	5.032	2.5×10^{-4}
GtsRNA-Gly-GCC	3.092	2.6×10^{-3}	4.282	4.0×10^{-3}	6.375	9.7×10^{-7}
GtsRNA-Gly-TCC	3.059	2.9×10^{-3}	3.937	1.4×10^{-2}	6.148	2.6×10^{-6}
GtsRNA-His-GTG	2.580	1.1×10^{-2}	3.577	4.8×10^{-2}	4.316	3.8×10^{-3}
GtsRNA-Leu-TAA	2.602	1.1×10^{-2}	4.005	1.1×10^{-2}	4.611	1.3×10^{-3}
GtsRNA-Lys-TTT	3.380	1.1×10^{-3}	4.786	5.9×10^{-4}	5.229	1.1×10^{-4}
GtsRNA-Met-CAT	2.428	1.7×10^{-2}	4.046	9.4×10^{-3}	4.965	3.2×10^{-4}
GtsRNA-Pro-AGG	2.954	4.0×10^{-3}	4.512	1.7×10^{-3}	5.590	2.6×10^{-5}
GtsRNA-Pro-CGG	2.967	3.8×10^{-3}	4.520	1.6×10^{-3}	5.590	2.6×10^{-5}
GtsRNA-Pro-TGG	2.898	4.7×10^{-3}	4.454	2.1×10^{-3}	5.446	4.8×10^{-5}
GtsRNA-Ser-GCT	3.437	8.9×10^{-4}	3.878	1.7×10^{-2}	4.191	6.0×10^{-3}
GtsRNA-Val-TAC	3.470	8.0×10^{-4}	4.942	3.2×10^{-4}	4.904	4.1×10^{-4}
MtsRNA-His-GTG	3.004	3.4×10^{-3}	3.714	3.0×10^{-2}	3.832	2.1×10^{-2}
MtsRNA-Leu-TAA	2.926	4.3×10^{-3}	3.756	2.6×10^{-2}	4.791	6.4×10^{-4}
MtsRNA-Lys-TTT	3.309	1.3×10^{-3}	3.716	3.0×10^{-2}	4.396	2.8×10^{-3}
MtsRNA-Thr-TGT	3.047	3.0×10^{-3}	3.614	4.2×10^{-2}	4.929	3.7×10^{-4}

Note: The *t*-statistics and *P*-values were computed using a linear model controlling for age and sex. The *P*-value adjustment was performed using *Bonferroni* correction.
